# Oral Epithelial Dysplasia in Tobacco Non-habitués: A Case Report and Review of Literature

**DOI:** 10.7759/cureus.47362

**Published:** 2023-10-20

**Authors:** Anupama Mukherjee, Twyla Ferrao, Anita E Spadigam, Anita Dhupar

**Affiliations:** 1 Oral and Maxillofacial Pathology, Goa Dental College and Hospital, Bambolim, IND

**Keywords:** non-habitués, non-tobacco use, oral epithelial dysplasia, hpv-negative, etiology of dysplasia, molecular pathogenesis, malignant transformation

## Abstract

Oral potentially malignant disorders (OPMDs) encompass a diverse group of clinical lesions, which, on histopathological evaluation, may reveal features of hyperplasia, oral epithelial dysplasia (OED), or even early invasive squamous cell carcinoma. OEDs are often perceived to be associated with a deleterious habit such as tobacco chewing. It has emerged that OEDs may occur even in the absence of a tobacco habit and could be attributed to factors such as trauma, chronic inflammation, and inherent genetic aberrations. Authors have reported a preponderance of such lesions in young females, particularly at sites distinct from those noted in habitués. Additionally, the probability of malignant transformation of OED has been reported to be higher in non-habitués as compared to habitués when lesions are left unaddressed. There remains a paucity of data regarding the exact molecular basis, behavior, and response to treatment of OED among tobacco non-habitués. In view of the increasing number of oral lesions demonstrating epithelial dysplasia in the absence of exposure to significant risk factors, we highlight the scenario with a case.

A 39-year-old female, non-habitué, presented with a non-scrapable, white lesion on the maxillary buccal gingiva. Incisional biopsy revealed features of moderate epithelial dysplasia that, on further evaluation of the excisional specimen, confirmed features of severe epithelial dysplasia. Genotyping for human papillomavirus (HPV) was carried out to assess the presence of high-risk HPV strains (16, 18, 31, 33, 35, 39, 45, 51, 52, 56, 58, 59, 66, and 68), which are usually associated with OED and/or oral squamous cell carcinomas (OSCCs) in non-habitués. A comprehensive review of various tissue and molecular factors, which play a key role in the pathophysiology of non-habit-associated OED has been illustrated in this report. While the etiological focus of OPMDs is often directed toward deleterious habits and exposure to carcinogens, it is essential to be vigilant for this entity even among non-habitués. A meticulous screening of the oral cavity, for all patients, shall facilitate the prevention and early diagnosis of OED, particularly in individuals not exposed to habit-forming risk factors.

## Introduction

Oral squamous cell carcinoma (OSCC) is often preceded by clinical lesions that are collectively referred to as oral potentially malignant disorders (OPMDs). An OPMD is defined as “a group of disorders of varying etiologies, usually tobacco, characterized by mutagen-associated, spontaneous, or hereditary alterations or mutations in the genetic material of oral epithelial cells with or without clinical and histomorphological alterations that may lead to oral squamous cell carcinoma transformation” [1]. The worldwide prevalence rate of OPMDs ranges from 1% to 5% [2]. While categorization as an OPMD is a clinical diagnosis, histological evaluation may reveal a diagnosis that ranges from epithelial hyperplasia with or without hyperkeratosis to oral epithelial dysplasia (OED), to even oral squamous cell carcinoma (OSCC). OED is characterized by cytological and architectural alterations reflecting the loss of normal maturation and stratification of surface epithelium [3,4]. The risk of malignant transformation of oral epithelial dysplasia has been stated to progressively increase across the grades of OED [2].

While mild/moderate dysplasias have a 4%-11% rate of malignant transformation, severe epithelial dysplasia may transform into frank carcinoma in 2%-35% of cases [5]. The occurrence of OED is conventionally regarded as a result of exposure to chemical carcinogens such as tobacco and/or alcohol, in a dose- and time-dependent manner [6]. Numerous authors have recognized that in the absence of a habit history, it is often young females who present with oral lesions of a dysplastic nature and even oral squamous cell carcinoma [7-9]. Such a trend highlights the need to recognize and address the alternate risk factors and drivers for non-habit-related OED in an attempt to facilitate early diagnosis and prevent malignant transformation in this subset of oral epithelial dysplasia [10].

## Case presentation

**Figure 1:**
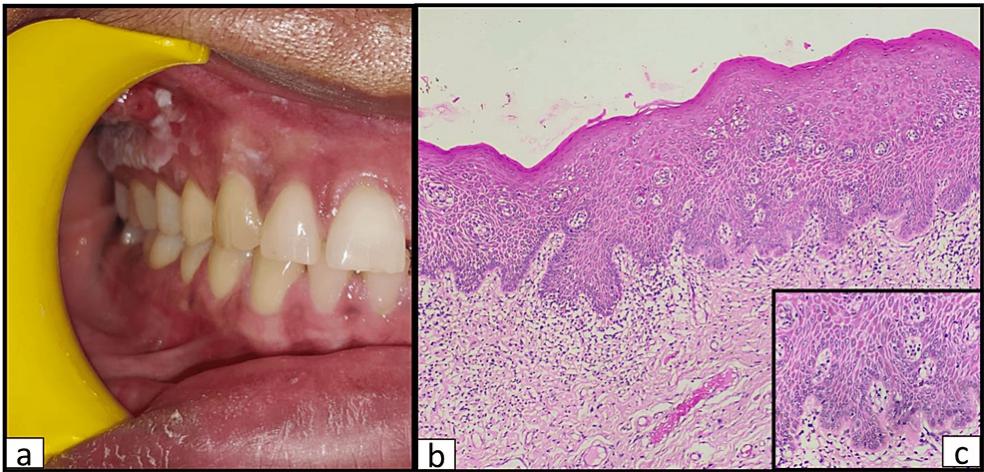
Clinical appearance of the lesion with corresponding histopathological features at incisional biopsy (a) Clinical appearance of the non-scrapable, white plaque restricted to the attached gingiva in relation to 13 extending up to the mesial aspect of 17 and extending into the buccal vestibule in a few areas. (b) Photomicrograph (4×) of hematoxylin and eosin-stained section showing stratified squamous parakeratinized epithelium with hyperkeratosis. The epithelium shows features of moderate epithelial dysplasia, along with a mild to moderate chronic inflammatory cell infiltrate. (c) Photomicrograph (10×) of hematoxylin and eosin-stained section showing features of dysplasia such as irregular epithelial stratification, loss of polarity of basal cells, few individual cell keratinization, altered nuclear-cytoplasmic ratio, and nuclear hyperchromasia along with nuclear and cellular pleomorphism.

A 39-year-old female presented to the department of oral and maxillofacial pathology with a chief complaint of a white lesion, which was occasionally accompanied by a burning sensation, on the right maxillary gingiva for 15 days. Upon anamnesis, a positive history of toothbrush trauma 20 days prior was noted. The patient also had a history of hypertension for one year and was being treated for the same. There was no history of tobacco usage, other deleterious habits, pernicious habits, or the presence of a similar oral lesion in the past. On examination, a white, non-scrapable lesion measuring 3.8 cm × 1 cm was noted on the attached gingiva, in relation to 13 to 17. The marginal gingiva in relation to these teeth remained spared of the lesion, which partially extended into the right maxillary buccal vestibule (Figure 1a).

**Figure 2:**
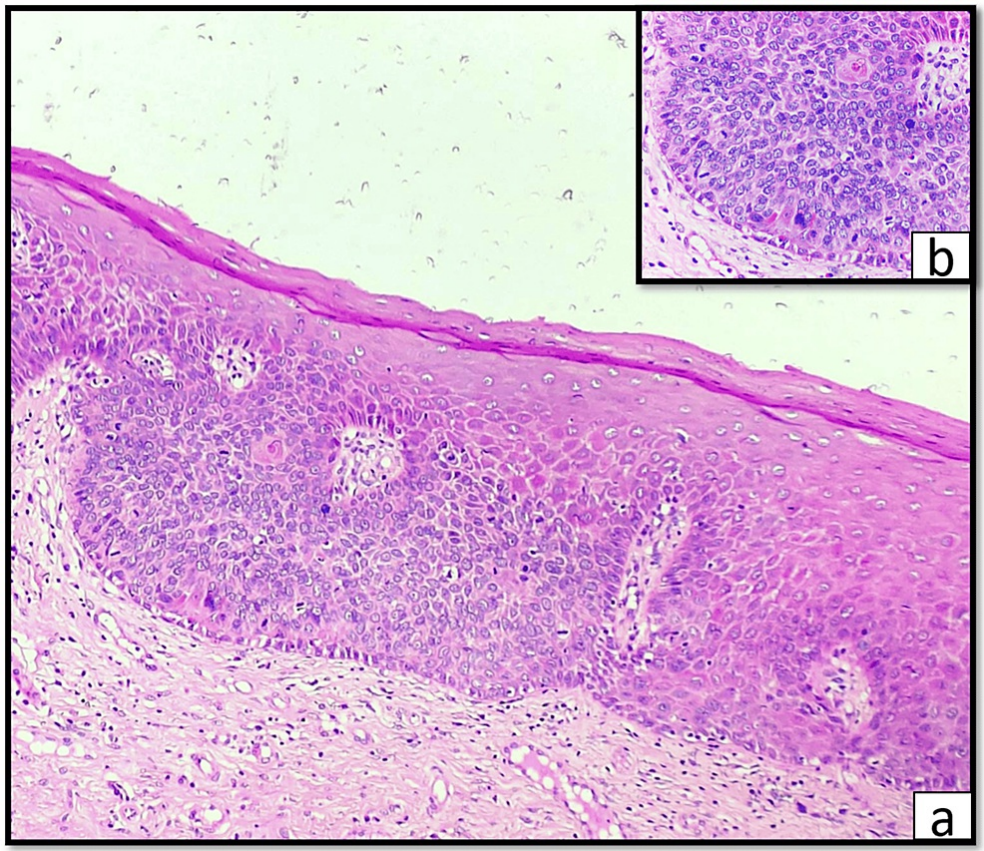
Histopathological evaluation of the excised specimen of the gingival lesion showing severe epithelial dysplasia (a) Photomicrograph (10×) of hematoxylin and eosin-stained section showing stratified squamous parakeratinized epithelium showing hyperplasia, hyperkeratosis, irregular epithelial stratification, multiple patterns (basaloid and keratinized) of dysplasia in different regions of the epithelium, loss of polarity of basal cells, altered nuclear-cytoplasmic ratio, nuclear and cellular pleomorphism, hyperchromatic nuclei, keratin pearl formation, and abnormal mitotic figures. (b) Photomicrograph (10×) of hematoxylin and eosin-stained section showing irregular epithelial stratification, loss of polarity of basal cells, altered nuclear-cytoplasmic ratio, nuclear and cellular pleomorphism, hyperchromatic nuclei, keratin pearl formation, and abnormal mitotic figures.

No regional lymph nodes were palpable. A provisional diagnosis of leukoplakia was assigned, and as part of the diagnostic workup, hematological assessment, exfoliative cytology, and an incisional biopsy were carried out. The hematological assessment revealed no abnormality. Evaluation of the PAP-stained exfoliative smear demonstrated an unremarkable class II cytology with no trace of candida. On incisional biopsy and routine histopathological assessment, features such as irregular stratification, loss of polarity and organization of basal cells, nuclear hyperchromatism, altered nuclear-cytoplasmic ratio, and nuclear and cellular pleomorphism were noted to involve the lower and middle third of the epithelium (Figure 1b, 1c). A diagnosis of moderate epithelial dysplasia was agreed upon (based on 2022 WHO criteria). The underlying connective tissue demonstrated collagen fibers, fibroblasts, and areas of mild to moderate chronic inflammatory cell infiltration (Figure 1b). A treatment plan was formulated, which included pharmacological management using antioxidants and complete excision of the lesion. A strict follow-up regimen was formulated with evaluation every month for the first three months, following which at six and 12 months. Histopathological examination of the excised specimen revealed features concurrent with a diagnosis of severe epithelial dysplasia (Figure 2a, 2b).

In view of the absence of a habit history, the patient was evaluated for human papillomavirus (HPV) using polymerase chain reaction (PCR). Genotyping revealed the absence of high-risk strains of HPV (16, 18, 31, 33, 35, 39, 45, 51, 52, 56, 58, 59, 66, and 68). Therefore, the above case draws attention toward the alternate etiological factors of OED, possibly trauma-induced chronic inflammation, and its impact on the overlying epithelium.

## Discussion

Prognostication of oral epithelial dysplasia (OED) continues to pose a challenge due to the subjective nature of its assessment, along with the lack of definitive predictive markers. While numerous grading systems have been proposed, so far, none have considered the etiological factors or the host (inflammatory) response associated with the lesion. The existing approach of assessment could be attributed to the common conjecture that OED and OSCC are a sequel of chemical carcinogenesis.

In 1863, Virchow endorsed the postulation earlier made by Galenus, associating inflammation and carcinogenesis [11]. Subsequently, Dvorak revealed that tumor tissues resembled wounds since both were composed of similar stromal cell types and demonstrated similar tissue responses; thus, tumors are often regarded as non-healing wounds [11]. In 1970, Prehn proposed the concept of inflammation-associated carcinogenesis, citing the occurrence of malignant tumors in individuals who had not been exposed to conventional risk factors. The malignant transformation rate for OED (among habitués and non-habitués) varies from 1.4% to 36%, of which non-smokers are 7.1 times more likely to undergo progression and transformation [2,11-13]. Non-habitués also present with lesions that are clinically categorized as OPMDs and could be attributed to chronic trauma/immune dysregulation and chronic inflammation. Clinical evaluation provides vital predictive information. Surface alterations such as a granular or verruciform appearance have demonstrated a malignant transformation potential of 4%-15%, while the homogenous, thick leukoplakia show transformation in nearly 1%-7% of cases [2,3,13,14]. Hence, the suspicion of an OPMD should arise in both habitués and non-habitués when changes in surface texture, loss of surface integrity, color, size, contour, or mobility of intraoral or extraoral structures are noted [3].

**Figure 3:**
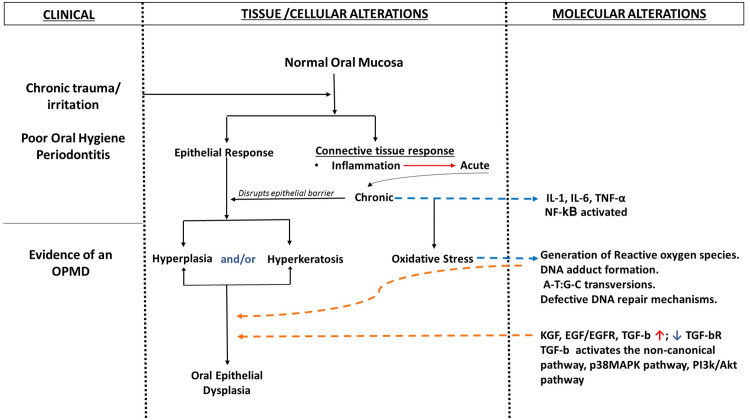
Illustration highlighting the proposed tissue and molecular interactions in inflammation-associated oral epithelial dysplasia Blue staggered arrows indicate molecular alterations as a result of processes occurring at a tissue/ cellular level. Orange staggered arrows indicate the tissue/cellular alterations as a result of distinct molecular processes. OPMD: oral potentially malignant disorder, IL: interleukin, TNF-α: tumor necrosis factor-α, NF-κB: nuclear factor kappa B, DNA: deoxyribonucleic acid, KGF: keratinocyte growth factor, EGF: epidermal growth factor, EGFR: epidermal growth factor receptor, TGF-b: transforming growth factor-beta, TGF-bR: transforming growth factor-beta receptor, MAPK: mitogen-activated protein kinase Image credits: Dr. Anupama Mukherjee and Dr. Twyla Ferrao

Data regarding a distinction between habit- and non-habit-associated OED is limited, and few studies have revealed a greater potential for malignant transformation among non-habitués. As reported by Rock et al. (2018), non-smokers were twice as likely to undergo malignant transformation as compared to smokers. The time for progression of OED to malignancy in non-smokers was reported to be faster as compared to smokers [8]. Jaber (2010) also reported a higher degree of malignant transformation in non-users as compared to users, although the long-term outcome of both groups was reported to be similar [10]. The absence of any known potential risk factor such as the habitual use of tobacco in a smoked or smokeless form hints toward specific molecular and genetic mechanisms that may trigger carcinogenesis in non-habitués. A clinical trigger such as trauma or chronic irritation results in early tissue responses such as hyperplasia and hyperkeratosis involving the epithelium, while inflammation in its acute and chronic forms are the key stromal responses. Sustained inflammation leads to the activation of nuclear factor kappa B (NF-κB) along with an increase in interleukin (IL)-1, IL-4, IL-6, and tumor necrosis factor (TNF)-α. These cytokines are key players in propagating oxidative stress in tissues, which further leads to DNA adduct formation, nucleotide transversions, and defective DNA repair mechanisms. The numerous molecular events along with the activation of the non-canonical transforming growth factor-beta (TGF-b) pathway, mitogen-activated protein kinase (MAPK) pathway, and PI3k/Akt pathway result in dysregulated cell proliferation, morphological alterations, and irregular tissue organization, recognized histopathologically as features of oral epithelial dysplasia (Figure 3) [11,15-18].

Inflammation-associated carcinogenesis has been reported as a phenomenon to account for the occurrence of malignancy in patients not exposed to conventional carcinogens. It implicates the role of chronic inflammation resulting in the recruitment of immune cells along with the generation of reactive oxygen and nitrogen species. The net oxidative stress results in DNA damage and mutations. The DNA repair mechanisms involved in maintaining an error-free genome have also been implicated to potentiate carcinogenesis when they fail to effectively proofread the replicating DNA [19]. Ho et al. (2012) highlighted risk factors that indicate a greater tendency of malignant transformation, of which idiopathic leukoplakia (i.e., in non-smokers), lesions noted in female patients, and a higher grade of the lesion are possible beacons for malignant transformation in non-habit-associated OED [3]. Authors have reported that clinical intervention at the stage of mild epithelial dysplasia often resolves the lesion, while success for moderate and severe dysplasia varies. While no standardized and universally accepted protocol for the management of dysplasia exists, it would be imperative to explore the management strategies in both scenarios as lesions in non-habitués have demonstrated a higher risk of malignant transformation as compared to those in habitués who have an overall higher risk of presenting with an OPMD [8].

## Conclusions

There is a paucity of data regarding the distinction between epithelial dysplasia in tobacco habitués and non-habitués. Speculations concerning the possible etiology or combination of etiological agents that precipitate OED in non-habitués need to be evaluated at a molecular and genetic level. It remains to be explored whether the occurrence of dysplasia among tobacco habitués and non-habitués differs merely in its pathogenesis or also in its behavior and outcome. Demographic variations in the presentation of non-habit-associated OED, such as its occurrence in younger individuals and the involvement of relatively uncommon sites such as the gingiva, warrant detailed anamnesis and a watchful screening of every patient. An effort to facilitate early detection and interception of dysplastic lesions in the unassuming population must be advocated.
